# Crystal structure of strontium dinickel iron orthophosphate

**DOI:** 10.1107/S205698901501779X

**Published:** 2015-09-26

**Authors:** Said Ouaatta, Abderrazzak Assani, Mohamed Saadi, Lahcen El Ammari

**Affiliations:** a Laboratoire de Chimie du Solide Appliquée, Faculté des Sciences, Université Mohammed V, Avenue Ibn Battouta, BP 1014, Rabat, Morocco

**Keywords:** crystal structure, transition metal phosphates, solid-state reaction synthesis, SrNi_2_Fe(PO_4_)_3_, α-chromium phosphate

## Abstract

The title compound, SrNi_2_Fe(PO_4_)_3_, synthesized by solid-state reaction, crystallizes in an ordered variant of the α-CrPO_4_ structure. In the asymmetric unit, two O atoms are in general positions, whereas all others atoms are in special positions of the space group *Imma*: the Sr cation and one P atom occupy the Wyckoff position 4*e* (*mm2*), Fe is on 4*b* (*2/m*), Ni and the other P atom are on 8*g* (*2*), one O atom is on 8*h* (*m*) and the other on 8*i* (*m*). The three-dimensional framework of the crystal structure is built up by [PO_4_] tetra­hedra, [FeO_6_] octa­hedra and [Ni_2_O_10_] dimers of edge-sharing octa­hedra, linked through common corners or edges. This structure comprises two types of layers stacked alternately along the [100] direction. The first layer is formed by edge-sharing octa­hedra ([Ni_2_O_10_] dimer) linked to [PO_4_] tetra­hedra *via* common edges while the second layer is built up from a strontium row followed by infinite chains of alternating [PO_4_] tetra­hedra and FeO_6_ octa­hedra sharing apices. The layers are held together through vertices of [PO_4_] tetra­hedra and [FeO_6_] octa­hedra, leading to the appearance of two types of tunnels parallel to the *a*- and *b*-axis directions in which the Sr cations are located. Each Sr cation is surrounded by eight O atoms.

## Abstract 2

The transition metal orthophosphates Sr*M*_2_Fe(PO_4_)_3_ (*M* = Co, Ni) crystallize in an α-CrPO_4_-type structure. The chains characterizing this structure are then built up from [Ni_2_O_10_] units alternating with [PO_4_] tetra­hedra and [FeO_6_] octa­hedra. The structure is nearly the same as that observed in *M*Mn^II^_2_Mn^III^(PO_4_)_3_ (*M* = Pb, Sr, Ba).

## Chemical context   

Phosphates with the alluaudite (Moore, 1971 ▸) and α-CrPO_4_ (Attfield *et al.*, 1988 ▸) crystal structures have attracted great inter­est due to their potential applications as battery electrodes (Trad *et al.*, 2010 ▸; Kim *et al.*, 2014 ▸; Huang *et al.*, 2015 ▸). In the last decade, our inter­est has focused on those two phosphate derivatives and we have succeeded in synthesizing and structurally characterizing new phosphates such as Na_2_Co_2_Fe(PO_4_)_3_ (Bouraima *et al.*, 2015 ▸) and Na_1.67_Zn_1.67_Fe_1.33_(PO_4_)_3_ (Khmiyas *et al.*, 2015 ▸) with the alluaudite structure type, and *M*Mn^II^_2_Mn^III^(PO_4_)_3_ (*M* = Pb, Sr, Ba) (Alhakmi *et al.* (2013*a* ▸,*b* ▸; Assani *et al.*, 2013 ▸) which belongs to the α-CrPO_4_ structure type. In the same context, our solid-state chemistry investigations within the ternary system *M*O–*M*′O–NiO–P_2_O_5_ (*M* and *M*′ are divalent cations), have led to the synthesis of the title compound SrNi_2_Fe(PO_4_)_3_ which has a related α-CrPO_4_ structure.

## Structural commentary   

**Figure 1:**
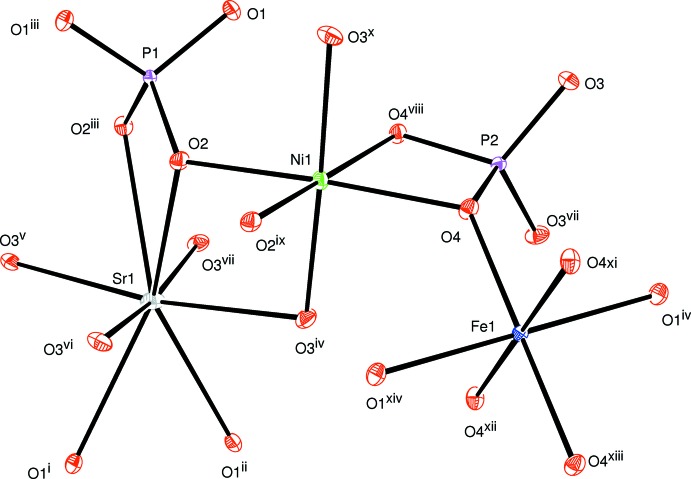
The principal building units in the structure of the title compound. Displacement ellipsoids are drawn at the 50% probability level. [Symmetry codes: (i) −*x* + 1, −*y* + 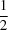, *z* − 1; (ii) *x*, *y*, *z* − 1; (iii) −*x* + 1, −*y* + 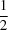, *z*; (iv) −*x* + 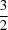, −*y* + 1, *z* − 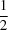; (v) *x* − 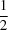, *y* − 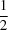, *z* − 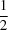; (vi) −*x* + 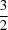, *y* − 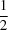, *z* − 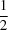; (vii) *x* − 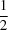, −*y* + 1, *z* − 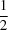; (viii) −*x* + 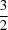, *y*, −*z* + 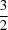; (ix) −*x* + 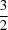, −*y* + 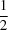, −*z* + 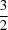; (*x*) *x*, −*y* + 1, −*z* + 2; (xi) −*x* + 2, *y*, *z*; (xii) *x*, −*y* + 1, −*z* + 1; (xiii) −*x* + 2, −*y* + 1, −*z* + 1; (xiv) *x* + 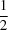, *y*, −*z* + 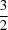.]

**Figure 2:**
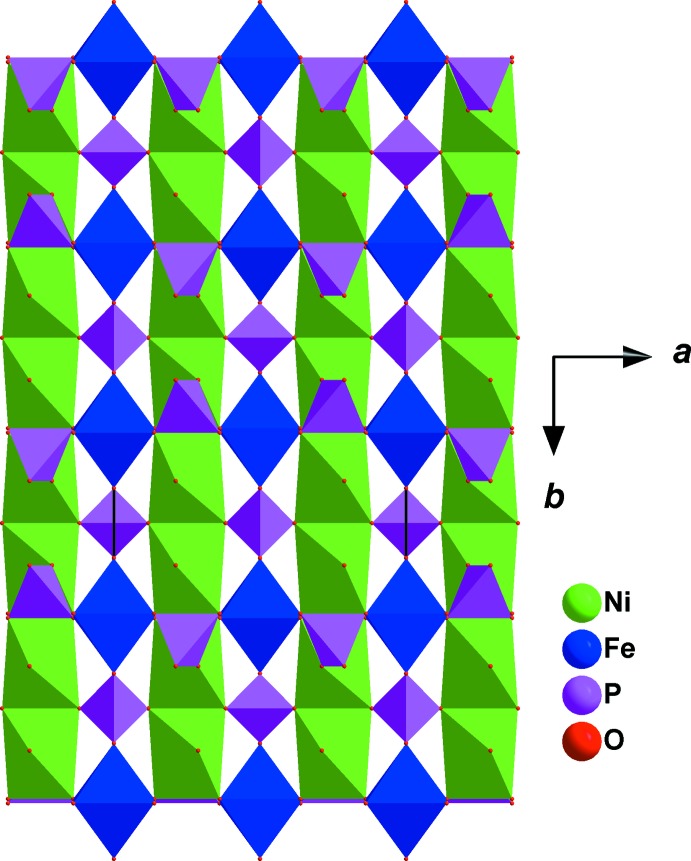
Stacking along [100] of layers building the crystal structure of SrNi_2_Fe(PO_4_)_3_.

**Figure 3:**
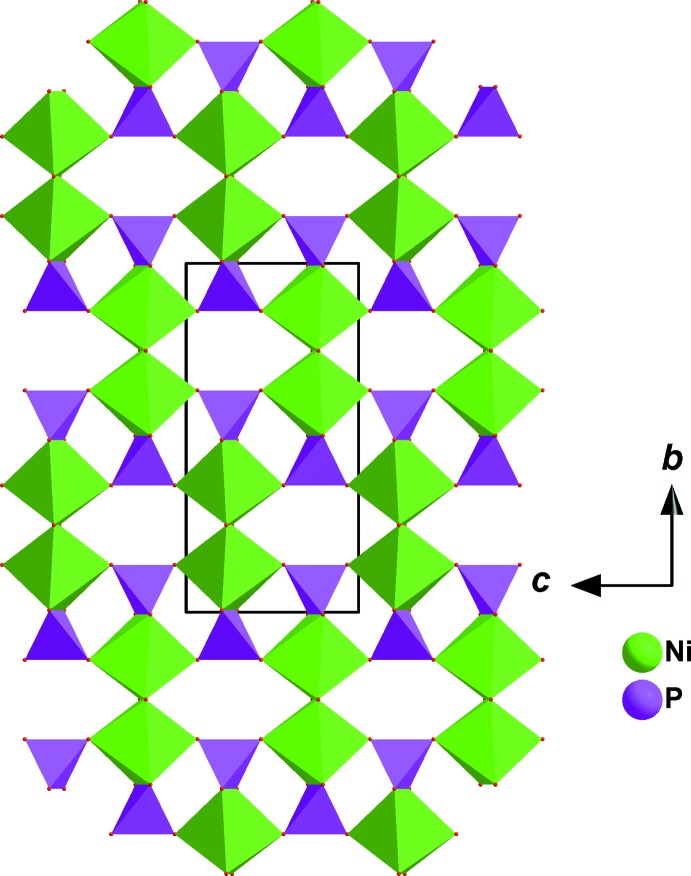
View along the *a* axis of a layer resulting from the connection of [Ni_2_O_10_] dimers and [PO_4_] tetra­hedra *via* common edges or vertices. Sr cations are omitted.

**Figure 4:**
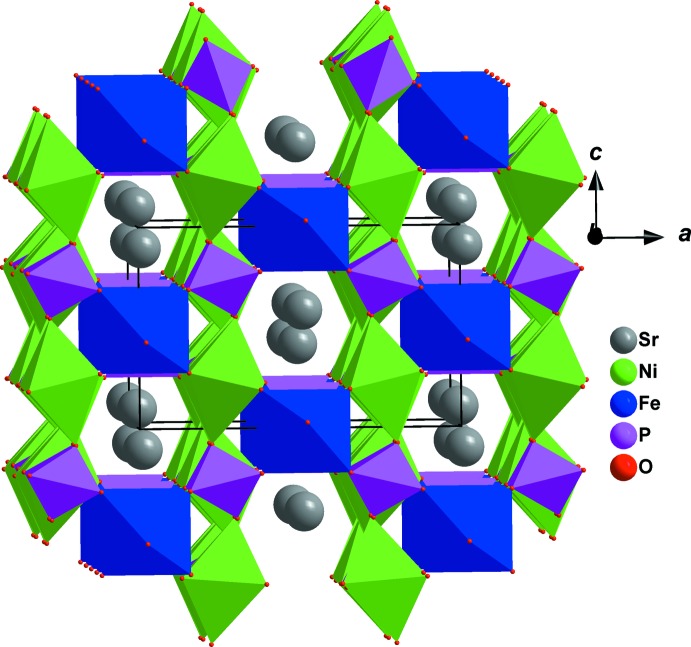
Polyhedral representation of the crystal structure of SrNi_2_Fe(PO_4_)_3_ showing tunnels running along [010].

The crystal structure of the title phosphate is formed by [PO_4_] tetra­hedra linked to [NiO_6_] and [FeO_6_] octa­hedra, as shown in Fig. 1 ▸. The octa­hedral environment of iron is more distorted than that of nickel (see Table 1 ▸). In this model, bond-valence-sum calculations (Brown & Altermatt, 1985 ▸) for Sr^2+^, Ni^2+^, Fe^3+^, P1^5+^and P2^5+^ ions are as expected, *viz.* 1.88, 1.95, 2.91, 5.14 and 5.01 valence units, respectively. Atoms Sr1 and P1 occupy Wyckoff positions 4*e* (*mm2*), Fe1 is on 4*b* (*2/m*), Ni1 and P2 are on 8*g* (*2*), O1 is on 8h (*m*) and O2 is on 8i (*m*)·The three-dimensional network of the crystal structure is composed of two types of layers parallel to (100), as shown in Fig. 2 ▸. The first layer is built up from two adjacent edge-sharing octa­hedra ([Ni_2_O_10_] dimers) whose ends are connected to [PO_4_] tetra­hedra by a common edge or vertex (Fig. 3 ▸). The second layer is formed by an Sr row followed by infinite chains of alternating [PO_4_] tetra­hedra and [FeO_6_] octa­hedra sharing apices. These two types of layers are linked together by common vertices of [PO_4_] tetra­hedra, forming a three-dimensional framework which delimits two types of tunnels running along the *a-* and *b*-axis directions in which the Sr cations are located with eight neighbouring O atoms (Fig. 4 ▸). The structure of the title compound is isotypic to that of *M*Mn^II^_2_Mn^III^(PO_4_)_3_ (*M* = Pb, Sr, Ba).

## Database Survey   

It is inter­esting to compare the crystal structure of α-CrPO_4_ (Glaum *et al.*, 1986 ▸) with that of the title compound. Both phosphates crystallize in the ortho­rhom­bic system in the space group *Imma*. Moreover, their unit-cell parameters are nearly the same despite the difference between their chemical formulas. In the structure of α-CrPO_4_, the Cr^3+^ and P^5+^ cations occupy four special positions and the three-dimensional concatenation of [PO_4_] tetra­hedra and [CrO_6_] octa­hedra allows the formation of empty tunnels along the *b*-axis direction. We can write the formula of this phosphate as follows: *LL*′(Cr1)_2_Cr2(PO_4_)_3_, and in the general case, *AA*′*M*_2_ *M*′(PO_4_)_3_ where *L* and *L*′ represent the two empty tunnels sites, while *M* and *M*′ correspond to the trivalent cation octa­hedral sites. This model is in accordance with that of the alluaudite structure which is represented by the general formula *A*A′*M*_2_ *M*′(*X*O_4_)_3_ and is closely related to the α-CrPO_4_ structure (*A* and *A*′ represent the two tunnels sites which can be occupied by either mono- or divalent medium sized cations, while the *M* and *M*′ octa­hedral sites are generally occupied by transition metal cations). Accordingly, the substitution of Cr1 or Cr2 by a divalent cation requires charge compensation by a monovalent cation that will occupy the tunnel. Two very recently reported examples are Na_2_Co_2_Fe(PO_4_)_3_ and NaCr_2_Zn(PO_4_)_3_, which were characterized by X-ray diffraction, IR spectroscopy and magnetic measurements (Souiwa *et al.*, 2015 ▸). The replacement of Cr1 by a divalent cation involves an amendment of the charge by a divalent cation as in the present case, SrNi_2_Fe(PO_4_)_3_, which is a continuation of our previous work, namely *M*Mn^II^_2_Mn^III^(PO_4_)_3_ (*M* = Pb, Sr, Ba).

## Synthesis and crystallization   

SrNi_2_Fe(PO_4_)_3_ was synthesized by a solid state reaction in air. Stoichiometric qu­anti­ties of strontium, nickel, and iron nitrates and 85 wt% phospho­ric acid were dissolved in water. The resulting solution was stirred without heating for 20 h and was, subsequently, evaporated to dryness. The obtained dry residue was homogenized in an agate mortar and then progressively heated in a platinum crucible up to 873 K. The reaction mixture was maintained at this temperature during 24 h before being heated to the melting point of 1373 K. The molten product was then cooled down slowly to room temperature at a rate of 5 K h^−1^ rate. Orange parallelepiped-shaped crystals of the title compound were thus obtained.

## Refinement   

Crystal data, data collection and structure refinement details are summarized in Table 2 ▸. The highest peak and the deepest hole in the final Fourier map are at 0.72 and 0.80 Å from Sr1 and P1, respectively. 

## Supplementary Material

Crystal structure: contains datablock(s) I. DOI: 10.1107/S205698901501779X/pj2022sup1.cif

Structure factors: contains datablock(s) I. DOI: 10.1107/S205698901501779X/pj2022Isup2.hkl

CCDC reference: 1426730

Additional supporting information:  crystallographic information; 3D view; checkCIF report

## Figures and Tables

**Table 1:** Selected bond lengths ()

Sr1O1^i^	2.6390(13)	Fe1O4	1.9703(8)
Sr1O2	2.6477(12)	Fe1O1^ii^	2.0751(12)
Sr1O3^ii^	2.6662(9)	P1O1	1.5239(12)
Ni1O4	2.0561(8)	P1O2	1.5514(12)
Ni1O2	2.0612(8)	P2O3	1.5223(9)
Ni1O3^iii^	2.0953(9)	P2O4	1.5722(9)

Symmetry codes: (i) 
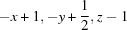
; (ii) 
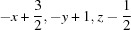
; (iii) 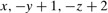.

**Table 2:** Experimental details

Crystal data	
Chemical formula	SrNi_2_Fe(PO_4_)_3_
*M*_r_	545.80
Crystal system, space group	Orthorhombic, *I* *m* *m* *a*
Temperature (K)	296
*a*, *b*, *c* ()	10.3881(11), 13.1593(13), 6.5117(7)
*V* (^3^)	890.15(16)
*Z*	4
Radiation type	Mo *K*
(mm^1^)	12.34
Crystal size (mm)	0.31 0.25 0.19

Data collection	
Diffractometer	Bruker X8 APEX
Absorption correction	Multi-scan (*SADABS*; Bruker, 2009 ▸)
*T*_min_, *T*_max_	0.504, 0.748
No. of measured, independent and observed [*I* > 2(*I*)] reflections	8211, 1112, 1095
*R*_int_	0.024
(sin /)_max_ (^1^)	0.820

Refinement	
*R*[*F* ^2^ > 2(*F* ^2^)], *wR*(*F* ^2^), *S*	0.015, 0.041, 1.20
No. of reflections	1112
No. of parameters	54
_max_, _min_ (e ^3^)	0.92, 0.57

Computer programs: *APEX2* and *SAINT* (Bruker, 2009 ▸), *SHELXS97* and *SHELXL97* (Sheldrick, 2008 ▸), *ORTEP-3 for Windows* (Farrugia, 2012 ▸), *DIAMOND* (Brandenburg, 2006 ▸), and *publCIF* (Westrip, 2010 ▸).

## supplementary crystallographic information

### Crystal data

**Table:**

SrNi_2_Fe(PO_4_)_3_	*D*_x_ = 4.073 Mg m^−^^3^
*M**_r_* = 545.80	Mo *K*α radiation, λ = 0.71073 Å
Orthorhombic, *I**m**m**a*	Cell parameters from 1112 reflections
*a* = 10.3881 (11) Å	θ = 3.1–35.6°
*b* = 13.1593 (13) Å	µ = 12.34 mm^−^^1^
*c* = 6.5117 (7) Å	*T* = 296 K
*V* = 890.15 (16) Å^3^	Parallelepiped, orange
*Z* = 4	0.31 × 0.25 × 0.19 mm
*F*(000) = 1044	

### Data collection

**Table:**

Bruker X8 APEX Diffractometer	1112 independent reflections
Radiation source: fine-focus sealed tube	1095 reflections with *I* > 2σ(*I*)
Graphite monochromator	*R*_int_ = 0.024
φ and ω scans	θ_max_ = 35.6°, θ_min_ = 3.1°
Absorption correction: multi-scan (*SADABS*; Bruker, 2009)	*h* = −17→17
*T*_min_ = 0.504, *T*_max_ = 0.748	*k* = −21→21
8211 measured reflections	*l* = −9→10

### Refinement

**Table:**

Refinement on *F*^2^	0 restraints
Least-squares matrix: full	*w* = 1/[σ^2^(*F*_o_^2^) + (0.0211*P*)^2^ + 1.0433*P*] where *P* = (*F*_o_^2^ + 2*F*_c_^2^)/3
*R*[*F*^2^ > 2σ(*F*^2^)] = 0.015	(Δ/σ)_max_ = 0.001
*wR*(*F*^2^) = 0.041	Δρ_max_ = 0.92 e Å^−^^3^
*S* = 1.20	Δρ_min_ = −0.57 e Å^−^^3^
1112 reflections	Extinction correction: *SHELXL*, Fc^*^=kFc[1+0.001xFc^2^λ^3^/sin(2θ)]^-1/4^
54 parameters	Extinction coefficient: 0.0040 (3)

### Special details

**Table:**

Geometry. All e.s.d.'s (except the e.s.d. in the dihedral angle between two l.s. planes) are estimated using the full covariance matrix. The cell e.s.d.'s are taken into account individually in the estimation of e.s.d.'s in distances, angles and torsion angles; correlations between e.s.d.'s in cell parameters are only used when they are defined by crystal symmetry. An approximate (isotropic) treatment of cell e.s.d.'s is used for estimating e.s.d.'s involving l.s. planes.

### Fractional atomic coordinates and isotropic or equivalent isotropic displacement parameters (Å^2^)

**Table:**

	*x*	*y*	*z*	*U*_iso_*/*U*_eq_	
Sr1	0.5000	0.2500	0.40652 (3)	0.00832 (6)	
Ni1	0.7500	0.36678 (2)	0.7500	0.00507 (6)	
Fe1	1.0000	0.5000	0.5000	0.00365 (7)	
P1	0.5000	0.2500	0.91246 (8)	0.00335 (9)	
P2	0.7500	0.57166 (3)	0.7500	0.00391 (8)	
O1	0.5000	0.34416 (9)	1.04869 (19)	0.00631 (18)	
O2	0.61817 (11)	0.2500	0.76678 (18)	0.00566 (18)	
O3	0.78842 (9)	0.63613 (6)	0.93417 (14)	0.00764 (14)	
O4	0.86173 (8)	0.49396 (6)	0.70676 (14)	0.00586 (13)	

### Atomic displacement parameters (Å^2^)

**Table:**

	*U*^11^	*U*^22^	*U*^33^	*U*^12^	*U*^13^	*U*^23^
Sr1	0.00864 (10)	0.01114 (10)	0.00518 (9)	0.000	0.000	0.000
Ni1	0.00501 (9)	0.00407 (9)	0.00613 (10)	0.000	0.00049 (6)	0.000
Fe1	0.00281 (12)	0.00403 (12)	0.00410 (12)	0.000	0.000	0.00015 (9)
P1	0.0033 (2)	0.0031 (2)	0.0037 (2)	0.000	0.000	0.000
P2	0.00410 (15)	0.00389 (15)	0.00374 (15)	0.000	0.00042 (10)	0.000
O1	0.0074 (4)	0.0049 (4)	0.0067 (4)	0.000	0.000	−0.0014 (4)
O2	0.0043 (4)	0.0063 (4)	0.0064 (4)	0.000	0.0017 (3)	0.000
O3	0.0095 (3)	0.0080 (3)	0.0055 (3)	−0.0019 (3)	0.0002 (3)	−0.0020 (2)
O4	0.0045 (3)	0.0056 (3)	0.0074 (3)	0.0005 (2)	0.0019 (3)	0.0005 (2)

### Geometric parameters (Å, º)

**Table:**

Sr1—O1^i^	2.6390 (13)	Fe1—O4^xi^	1.9703 (8)
Sr1—O1^ii^	2.6390 (13)	Fe1—O4^xii^	1.9703 (8)
Sr1—O2	2.6477 (12)	Fe1—O4^xiii^	1.9703 (8)
Sr1—O2^iii^	2.6477 (12)	Fe1—O4	1.9703 (8)
Sr1—O3^iv^	2.6662 (9)	Fe1—O1^xiv^	2.0751 (12)
Sr1—O3^v^	2.6662 (9)	Fe1—O1^iv^	2.0751 (12)
Sr1—O3^vi^	2.6662 (9)	P1—O1	1.5239 (12)
Sr1—O3^vii^	2.6662 (9)	P1—O1^iii^	1.5239 (12)
Ni1—O4^viii^	2.0561 (8)	P1—O2^iii^	1.5514 (12)
Ni1—O4	2.0561 (8)	P1—O2	1.5514 (12)
Ni1—O2	2.0612 (8)	P2—O3	1.5223 (9)
Ni1—O2^ix^	2.0612 (8)	P2—O3^viii^	1.5223 (9)
Ni1—O3^x^	2.0953 (9)	P2—O4	1.5722 (9)
Ni1—O3^iv^	2.0953 (9)	P2—O4^viii^	1.5722 (9)
			
O1^i^—Sr1—O1^ii^	56.01 (5)	O4—Ni1—O3^x^	92.39 (4)
O1^i^—Sr1—O2	141.47 (2)	O2—Ni1—O3^x^	93.49 (4)
O1^ii^—Sr1—O2	141.47 (2)	O2^ix^—Ni1—O3^x^	84.94 (4)
O1^i^—Sr1—O2^iii^	141.47 (2)	O4^viii^—Ni1—O3^iv^	92.39 (4)
O1^ii^—Sr1—O2^iii^	141.47 (2)	O4—Ni1—O3^iv^	89.31 (3)
O2—Sr1—O2^iii^	55.24 (5)	O2—Ni1—O3^iv^	84.94 (4)
O1^i^—Sr1—O3^iv^	108.88 (2)	O2^ix^—Ni1—O3^iv^	93.49 (4)
O1^ii^—Sr1—O3^iv^	78.22 (2)	O3^x^—Ni1—O3^iv^	177.91 (5)
O2—Sr1—O3^iv^	63.76 (3)	O4^xi^—Fe1—O4^xii^	180.0
O2^iii^—Sr1—O3^iv^	108.81 (3)	O4^xi^—Fe1—O4^xiii^	86.39 (5)
O1^i^—Sr1—O3^v^	78.22 (2)	O4^xii^—Fe1—O4^xiii^	93.61 (5)
O1^ii^—Sr1—O3^v^	108.88 (2)	O4^xi^—Fe1—O4	93.61 (5)
O2—Sr1—O3^v^	108.81 (3)	O4^xii^—Fe1—O4	86.39 (5)
O2^iii^—Sr1—O3^v^	63.76 (3)	O4^xiii^—Fe1—O4	180.00 (3)
O3^iv^—Sr1—O3^v^	172.25 (4)	O4^xi^—Fe1—O1^xiv^	93.70 (3)
O1^i^—Sr1—O3^vi^	78.22 (2)	O4^xii^—Fe1—O1^xiv^	86.30 (3)
O1^ii^—Sr1—O3^vi^	108.88 (2)	O4^xiii^—Fe1—O1^xiv^	86.30 (3)
O2—Sr1—O3^vi^	63.76 (3)	O4—Fe1—O1^xiv^	93.70 (3)
O2^iii^—Sr1—O3^vi^	108.81 (3)	O4^xi^—Fe1—O1^iv^	86.30 (3)
O3^iv^—Sr1—O3^vi^	68.39 (4)	O4^xii^—Fe1—O1^iv^	93.70 (3)
O3^v^—Sr1—O3^vi^	111.05 (4)	O4^xiii^—Fe1—O1^iv^	93.70 (3)
O1^i^—Sr1—O3^vii^	108.88 (2)	O4—Fe1—O1^iv^	86.30 (3)
O1^ii^—Sr1—O3^vii^	78.22 (2)	O1^xiv^—Fe1—O1^iv^	180.00 (7)
O2—Sr1—O3^vii^	108.81 (3)	O1—P1—O1^iii^	108.80 (10)
O2^iii^—Sr1—O3^vii^	63.76 (3)	O1—P1—O2^iii^	110.85 (3)
O3^iv^—Sr1—O3^vii^	111.05 (4)	O1^iii^—P1—O2^iii^	110.85 (3)
O3^v^—Sr1—O3^vii^	68.39 (4)	O1—P1—O2	110.85 (3)
O3^vi^—Sr1—O3^vii^	172.25 (4)	O1^iii^—P1—O2	110.85 (3)
O4^viii^—Ni1—O4	71.02 (5)	O2^iii^—P1—O2	104.61 (9)
O4^viii^—Ni1—O2	102.98 (3)	O3—P2—O3^viii^	112.25 (7)
O4—Ni1—O2	171.55 (4)	O3—P2—O4	108.06 (5)
O4^viii^—Ni1—O2^ix^	171.55 (4)	O3^viii^—P2—O4	114.51 (5)
O4—Ni1—O2^ix^	102.98 (3)	O3—P2—O4^viii^	114.51 (5)
O2—Ni1—O2^ix^	83.59 (5)	O3^viii^—P2—O4^viii^	108.06 (5)
O4^viii^—Ni1—O3^x^	89.31 (3)	O4—P2—O4^viii^	98.87 (6)

Symmetry codes: (i) −*x*+1, −*y*+1/2, *z*−1; (ii) *x*, *y*, *z*−1; (iii) −*x*+1, −*y*+1/2, *z*; (iv) −*x*+3/2, −*y*+1, *z*−1/2; (v) *x*−1/2, *y*−1/2, *z*−1/2; (vi) −*x*+3/2, *y*−1/2, *z*−1/2; (vii) *x*−1/2, −*y*+1, *z*−1/2; (viii) −*x*+3/2, *y*, −*z*+3/2; (ix) −*x*+3/2, −*y*+1/2, −*z*+3/2; (x) *x*, −*y*+1, −*z*+2; (xi) −*x*+2, *y*, *z*; (xii) *x*, −*y*+1, −*z*+1; (xiii) −*x*+2, −*y*+1, −*z*+1; (xiv) *x*+1/2, *y*, −*z*+3/2.
